# Use of continuous glucose monitoring in non-intensively managed type 2 diabetes: a Saudi Arabian consensus

**DOI:** 10.3389/fendo.2025.1738398

**Published:** 2026-01-12

**Authors:** Mohammed Almehthel, Abdulghani Al-Saeed, Fahad Al-Sabaan, Faisal Al-Malky, Hawazen Zarif, Lamya Al-Zubaidi, Mohammed E. Al-Sofiani, Omar Abdulaal, Reem Al Argan, Saud Al Sifri, Turki Al-Harbi, Raed Aldahash

**Affiliations:** 1 Obesity, Endocrinology and Metabolism Center, King Fahad Medical City, Riyadh, Saudi Arabia; 2 Division of Endocrinology, University of British Columbia, Vancouver, BC, Canada; 3 Department of Endocrinology and Diabetes, Diabetes Treatment Center, Prince Sultan Military Medical City, Riyadh, Saudi Arabia; 4 Security Forces Hospital, Riyadh, Saudi Arabia; 5 Diabetic Center, Al Noor Hospital, Makkah, Saudi Arabia; 6 Department of Medicine, Ministry of National Guard Health Affairs, Jeddah, Saudi Arabia; 7 College of Medicine, King Saud bin Abdulaziz University for Health Science, King Abdullah International Medical Research Center, Jeddah, Saudi Arabia; 8 Ministry of Health, Riyadh, Saudi Arabia; 9 Division of Endocrinology, Diabetes and Metabolism, College of Medicine, King Saud University, Riyadh, Saudi Arabia; 10 Division of Endocrinology, Diabetes & Metabolism, The Johns Hopkins University, Baltimore, MD, United States; 11 Internal Medicine Department, Prince Sultan Military Hospital, Madinah, Saudi Arabia; 12 Department of Internal Medicine, College of Medicine, Imam Abdulrahman Bin Faisal University, King Fahad Hospital of the University, Dammam, Eastern Province, Saudi Arabia; 13 Endocrinology Department, Alhada Armed Forces Hospital, Taif, Saudi Arabia; 14 King Saud bin Abdulaziz University for Health Science, Riyadh, Saudi Arabia; 15 King Abdullah International Medical Research Center, Riyadh, Saudi Arabia; 16 Department of Medicine, Ministry of National Guard – Health Affairs, Riyadh, Saudi Arabia

**Keywords:** continuous glucose monitoring, non-intensive, oral antidiabetics, Saudi Arabia, type 2 diabetes

## Abstract

**Background:**

Saudi Arabia has one of the highest prevalences of diabetes globally, with 16.4% of the population living with type 2 diabetes (T2D). While continuous glucose monitoring (CGM) is widely used for patients with type 1 diabetes, evidence suggests its benefits can extend to patients with T2D. The aim of this Delphi consensus was to provide a framework for the use of CGM in patients with T2D who are non-intensively managed in Saudi Arabia.

**Methods:**

An expert panel of ten adult endocrinology physicians, one internal medicine and diabetology specialist, and one family medicine physician was formed. Consensus generation was undertaken using Delphi methodology; a face-to-face expert meeting and literature review formed the basis of preliminary statements, which were further refined by the panel. Two rounds of voting were used to confirm the level of agreement to each statement.

**Results:**

Consensus was reached on 27 statements relating to the use of CGM in non-intensively managed T2D. Recommended patient profiles for continuous and intermittent use of CGM are provided, alongside general principles of CGM use and background statements.

**Conclusions:**

This consensus provides recommendations and summarizes local and international evidence as well as expert opinion regarding CGM use in patients with T2D. To expand the use of CGM into the wider population of T2D in Saudi Arabia and enable these individuals to benefit from the technology, a shift in healthcare services, education, and attitudes across the country is necessary.

## 1 Introduction

Diabetes is a chronic disease, characterized by elevated blood sugar levels resulting from insufficient insulin production or a lack of the body’s response to insulin (1). According to the 2021 International Diabetes Federation Diabetes Atlas 10th Edition, the global prevalence of diabetes in adults aged 20–79 years was 10.5% in 2021, with over 90% of these diabetes cases being type 2 diabetes (T2D) (1). Saudi Arabia has one of the highest prevalences of diabetes globally, with 4.3 million individuals aged 20–79 years living with diabetes in 2021, representing approximately 16.4% of the adult population (1). A systematic literature review of Saudi Arabian studies conducted by Jarrar et al, which included 19 studies published between 2000 and 2020, gave a pooled prevalence of 16.4% for T2D in all ages across Saudi Arabia (2).

Continuous glucose monitoring (CGM) is the continuous monitoring of glucose levels in the interstitial fluid via devices attached to the arm or abdomen (3). CGM devices are classed as either real-time CGM or intermittently scanned CGM (isCGM) depending on how the data is provided to the user (4). In contrast to self-monitoring of blood glucose (SMBG), which measures glucose levels at a single time-point, CGM is able to provide rate of change in glucose levels, supporting faster management and treatment of glycemic excursions (4). An additional key feature of CGM devices that offers important benefits over SMBG is the use of alarms, which alert the user if glucose levels fall outside of specified ranges (5). Furthermore, glucose data can also be shared directly with physicians, family members, and caregivers, which has shown to not only improve glycated hemoglobin (A1c) and reduce the occurrence of severe hypoglycemia events, but also contribute to improved well-being and less diabetes distress in users (6).

While CGM has been shown to be effective in patients with type 1 diabetes (T1D) and T2D (7), CGM is most commonly used in patients with T1D or patients with T2D on an intensive insulin regimen (8, 9). Recommendations for CGM in the management of diabetes have been outlined by the American Diabetes Association (ADA), American Association of Clinical Endocrinologists (AACE), UK National Institute for Health and Care Excellence (NICE), and Asia-Pacific (APAC) consensus recommendations.

ADA recommends CGM in all patients with diabetes using intensive insulin regimens of multiple daily injections (MDIs) or continuous subcutaneous insulin infusion as well as patients using basal insulin (10). AACE recommends CGM in all patients with diabetes using intensive insulin regimens as well as those with problematic hypoglycemia, all children or adolescents with T1D, women with gestational diabetes mellitus (GDM) using insulin, and pregnant women with either T1D or T2D on an intensive insulin regimen (11). Moreover, AACE specifies that CGM may also be recommended in patients with T2D on less intensive insulin therapy and women with GDM not using insulin (11). NICE recommends CGM in adults with either T1D or T2D who are using MDI and who measure their glucose levels at least eight times per day, those who require help with glucose monitoring due to disability or other condition, those with impaired glucose awareness, and patients with severe or recurrent hypoglycemia (12). Finally, the APAC consensus recommends CGM in all patients with diabetes using intensive insulin regimens, either as continuous or intermittent use. CGM may also be recommended in the following groups of patients with T2D using oral antidiabetics with or without basal or premix insulin: those with chronic kidney disease, those fasting during Ramadan, those with suboptimal glycemic control, frail patients ≥65 years, and pregnant women (13).

The aim of this Delphi consensus was to provide a framework for the use of CGM in patients with T2D who are non-intensively managed.

## 2 Materials and methods

The Delphi method was used for consensus generation. An expert panel consisting of 12 individuals was formed, including ten specialists in adult endocrinology, one internal medicine and diabetology specialist, and one family medicine physician. The panel were from 11 different institutions across Saudi Arabia, including Ministry of Health, Ministry of Interior, National Guard, Armed Forces, and referral hospitals.

A virtual face-to-face meeting was conducted on December 16, 2023 to discuss priority needs in the care of individuals with T2D who are not on intensive insulin therapy and how sensor-based technologies can help those individuals.

A detailed literature review was conducted to gather information on the use of CGM in patients with T2D who are non-intensively managed and on existing recommendations from guidelines. Search terms included “type 2 diabetes”, “continuous glucose monitoring”, “CGM”, “flash glucose monitoring”, “FGM”, “intermittently scanned continuous glucose monitoring”, “isCGM”, “non-intensive”, “non-insulin”, “basal insulin”, “pre-mixed insulin”, “biphasic insulin”, “glucose-lowering drugs”, “oral antidiabetics”, and “oral hypoglycemic agents”.

Based on the virtual meeting discussion and results of the literature review, preliminary statements were generated. The statements were then circulated for review by the expert panel and updated based on the panel’s comments. Subsequently, two rounds of voting were undertaken on the 27 consensus statements using the Delphi method. The panel ranked the consensus statements as “strongly agree”, “agree”, “disagree”, “strongly disagree”, or “neither agree nor disagree”. Consensus for a particular statement was achieved if ≥75% of the panel responded “strongly agree” or “agree” to a statement. If consensus was not reached on a particular statement, a virtual discussion was held to discuss the statements, statements were amended accordingly, and revoting was undertaken.

## 3 Results

Consensus was reached on 27 statements relating to the use of CGM in non-intensively managed type 2 diabetes.

### 3.1 Background

Experts agreed on all statements relating to background, with 100% level of agreement on all four statements (**Table 1**).

**Table 1:** Consensus statements on background.

#	Statement	Consensus (%)
1	T2D prevalence is high in Saudi Arabia.	100
2	Individuals with T2D in Saudi Arabia have high rates of comorbidities and diabetes-associated complications.	100
3	Intensively managed T2D patients are those on MDI of insulin, including premixed insulin or CSII.	100
4	Non-intensively managed T2D patients are those on basal insulin or non-insulin therapies.	100

CSII, continuous subcutaneous insulin infusion; MDI, multiple daily injections; T2D, type 2 diabetes.

High prevalence of T2D in the Kingdom of Saudi Arabia (KSA) has been reported in several studies. While the global prevalence of T2D is approximately 9% (1), the prevalence in KSA is much higher. In 2020, Mansour et al. reported a prevalence of 34.6% in adult Saudi nationals from Majmaah City (14). Additionally, in 2011, Al Daghri et al. reported a crude prevalence of 23.1% and an age-adjusted prevalence of 31.6% in Saudi nationals from Riyadh (15). A meta-analysis of 19 studies conducted by Jarrar et al. reported a pooled prevalence of 16.4% between 2000 and 2020 (2).

In addition to the high prevalence of T2D, the rate of comorbidities and diabetes-associated complications in KSA is also high. Al-Esawi et al. reported that 65.8% of males with T2D from Riyadh had diabetes complications, with cardiovascular complications being the most prevalent at 47.3% (16). Hypertension is one of the most common comorbidities, with reported prevalences of 38% to 70.5% (17–20). Coronary artery disease, stroke, dyslipidemia and obesity are further cardiovascular complications or risk factors with prevalences of 17.0-24% (19, 21), 3.7% (21), 66.5-69.0% (18, 22), 58.3% (18, 23, 24), respectively. Further complications include renal impairment (14.5%) (21), chronic kidney disease (8.7%) (18), diabetic nephropathy (10.8%) (25), retinopathy (6.25-88.1%) (26), neuropathy (20.3-37.4%) (21, 27–29). Furthermore, mental health conditions and sleep disorders are common among patients with T2D in KSA, with prevalences of 20-33.8% for depression, 38.3% for anxiety, 25.5% for stress, 22.3% for diabetes-related distress (22.3%), 63.7% for poor sleep and 44.3% for sleep apnea (17, 30–32). Finally, cancer has been associated with T2D in KSA. A regional meta-analysis calculated a population attributable risk of pancreatic cancer of 14.9%, colorectal cancer of 6.0%, breast cancer of 4.1%, and gastric cancer of 2.3% in patients with T2D from six studies in KSA (33).

Medical management of T2D can be classified as intensive or non-intensive. According to the Diabetes Control and Complications Trial, patients who were intensively managed were those taking multiple injections of insulin (three or more) per day or using an insulin pump along with monitoring of blood glucose levels four or more times per day (34). On the other hand, a non-intensive regimen has been described in a KSA study as patients using basal or premixed insulin, glucagon-like peptide 1 analogs, and/or oral antidiabetics (35).

### 3.2 General principles of CGM use in T2D

Experts agreed on all statements relating to the general principles of CGM use in T2D, with a level of agreement between 83.3% and 100% for the five statements (**Table 2**).

**Table 2:** Consensus statements on general principles of CGM use in T2D.

#	Statement	Consensus (%)
5	All patients with T2D can benefit from the use of CGM.	83.34
6	Clinical and real-world evidence in non-intensively insulin managed T2D demonstrates that CGM leads to a reduction in A1c, acute diabetes events, and hospitalizations.	91.67
7	Criteria for CGM use should be given irrespective of diabetes type.	83.34
8	Criteria for CGM use should be prioritized according to resources.	100
9	Specialists should engage with primary care physicians to empower them to prescribe CGM in T2D patients through improved knowledge, technical support, and materials to help onboard their patients.	100

CGM, continuous glucose monitoring; T2D, type 2 diabetes.

CGM is now established as a standard of care for T1D; however, there is also growing evidence supporting the use of CGM in everyday care of those with T2D (36). Several recent reviews have discussed the evidence for and benefits of using CGM in all patients with T2D, including those on non-intensive insulin or non-insulin therapies (36–39). CGM could enable those with T2D to improve their glycemic control early since continuous data generation is undertaken, enabling physicians to understand patterns of glucose variability, and through collaboration and feedback with patients, facilitate those with T2D to understand how their diet and activity can affect their glucose levels (40). In an observational study, weekly feedback by an endocrinology team in conjunction with CGM and an interdisciplinary team approach, led to an improvement in glycemic control in patients with T2D and suboptimal glycemic control (41). Use of CGM can also facilitate quick actions, such as treatment intensification, to be taken if a decline in control is detected (36). Evidence suggests there has been deterioration in glycemic control in patients with T2D using SMBG over time, with potential reasons being a lack of or insufficient glucose monitoring and the lack of success of SMBG in modifying patient behavior (39). Additionally, patients with T2D using non-intensive insulin regimens or sulfonylureas may be at risk of hypoglycemia, especially if SMBG is not performed adequately (39). Clinical and economic costs of inadequate control of diabetes are problematic, and this issue is likely to increase due to the rising prevalence of T2D across the globe (39). Therefore, CGM is instrumental in improving glycemic control and meeting the needs of all patients with T2D, and should be accessible to these patients (36, 37).

Studies conducted in Saudi Arabia have provided real-world evidence for the effectiveness of CGM in patients with T2D who are non-intensively managed. Al Hayek et al. conducted a retrospective cohort study of 93 patients with T2D from Prince Sultan Military Medical City, Riyadh, who were using basal or premixed insulin with or without non-insulin medications. The results of the analysis showed a significant reduction in HbA1c, from 8.3% at baseline to 8.1% (p<0.001) and 7.9% (p<0.001) in the first 90-day period and last 90-period of CGM use, respectively (35). The study also showed an improvement in CGM-captured glycemic markers, including a reduction in low glucose events, time below range (<70 mg/dL), time above range (>180–250 mg/dL), coefficient of variation, and average glucose, and an increase in time in range (70–180 mg/dL). These results remained significant when stratified into basal or premixed insulin users and non-insulin users (35). Furthermore, AlSifri et al. conducted a retrospective chart review of 1695 patients with diabetes from three hospitals in Taif, 545 of which were patients with T2D using oral antidiabetics and 314 of which were patients with T2D using basal insulin and oral antidiabetics (42). The study showed a mean reduction in HbA1c of 0.86% at 3 months, 1.27% at 6 months, and 1.67% at 12 months for patients with T2D on oral antidiabetics and a mean reduction of 0.94%, 1.33%, and 1.73% at 3, 6, and 12 months, respectively, for patients with T2D on basal insulin and oral antidiabetics after starting CGM (42).

Further studies in KSA have investigated the impact of CGM in all patients with T2D, regardless of the treatment used. Al Hayek et al. demonstrated a significant reduction in hypoglycemia episodes per month from 3.1 at baseline to 1.2 at 12 weeks for 105 patients with T2D from Prince Sultan Military Medical City, Riyadh (43). Additionally, an international systematic review of the literature in patients with T2D who were not using insulin reported a weighted mean difference of -0.31% in HbA1c in CGM users compared with SMBG, as well as significant reductions in glucose variation, glucose level, time below range, and time above range, and an increase in time in range and patient satisfaction (44).

Prioritization of CGM according to resources is an important aspect to consider in case of limitations in healthcare budgets. Challenges that may be faced by the Saudi healthcare system include health inequalities between urban and rural areas, shortage of healthcare professionals, and a deficit in preventive care (45). Resources for implementing CGM may be particularly problematic for primary care physicians, who treat the majority of patients with T2D (46). Additionally, the APAC consensus recommendations provide all recommendations according to resources (13).

The provision of improved knowledge, technical support, and materials to primary care physicians to help onboard their patients and to empower them to prescribe CGM is essential for patients with T2D. According to Ajjan et al, there is a need for a “paradigm shift” with regard to services, technology, education, and attitudes in order to support CGM in the management of T2D (36). Educational activities should include workshops and discussions regarding insurance issues and coverage (46). Primary care physicians should be guided on accessing glycemic data, the interpretation of CGM data, and safe and effective titration of diabetes therapies (47). Additionally, there is a need for support systems and expansion of databases pertaining to CGM use (47).

### 3.3 T2D patient profiles for continuous use of CGM

Experts agreed on all statements relating to T2D patient profiles for continuous use of CGM, with a level of agreement between 75% and 100% for the 10 statements (**Table 3**).

**Table 3:** Consensus statements on T2D patient profiles for continuous use of CGM.

#	Statement	Consensus (%)
10	Continuous use of CGM is strongly recommended for T2D patients at risk of hypoglycemia.	100
11	Continuous use of CGM is strongly recommended for T2D patients on basal insulin or premixed insulin.	75
12	Continuous use of CGM is recommended for elderly or frail T2D patients with multiple comorbidities.	91.67
13	Continuous use of CGM is recommended for youths or young adults with T2D.	100
14	Continuous use of CGM is recommended for patients with T2D who have dementia or high stroke risk.	91.67
15	Continuous use of CGM is recommended for T2D patients with cognitive or mental health impairment.	91.67
16	Continuous use of CGM is recommended for patients with T2D who received renal transplant.	100
17	Continuous use of CGM is recommended for patients with diabetes who have high cardiovascular risk.	100
18	Continuous use of CGM may be recommended for cystic fibrosis-related diabetes.	75
19	Continuous use of CGM may be recommended for T2D patients suffering from blindness.	91.67

CGM, continuous glucose monitoring; T2D, type 2 diabetes.

Risk of hypoglycemia in patients with T2D presents a challenge, particularly for elderly patients with numerous comorbidities, those with a history of hypoglycemia, and those with greater diabetes duration (48). In a study conducted in the Eastern Province of Saudi Arabia, the hypoglycemia prevalence was 12.5% in those with T2D, which was less than those with T1D (49). Nevertheless, since hypoglycemia unawareness is significant in patients with T2D in Saudi Arabia, with 50.5% of individuals with T2D using insulin in Madinah having hypoglycemia unawareness (50), reducing risk of hypoglycemia in these patients is essential. Additionally, during the holy month of Ramadan, hypoglycemia can be a challenge for fasting individuals with T2D. One study showed that 36% of individuals with T2D experienced at least one episode of hypoglycemia during Ramadan (51). Use of CGM in patients with T2D in KSA has been shown to reduce the frequency of hypoglycemia from 3.1 events per month at baseline to 1.2 events per month at 12 weeks (43).

Use of CGM in individuals with T2D using basal insulin or premixed insulin has been shown to reduce HbA1c in a Saudi study conducted over 1 year of CGM use (35). Additionally, a study conducted in the USA has shown that CGM use in patients with T2D 65 years or older taking basal insulin led to a decrease in time in range by a mean of 19% compared with SMBG (52). In patients with poorly controlled T2D using basal insulin, CGM led to a reduction in mean HbA1c from 9.1% to 8.0% after 8 months, a greater reduction than those using SMBG (53).

Elderly or frail individuals with T2D and multiple comorbidities are at greater risk of hypoglycemia. CGM has been shown to improve the detection of hypoglycemia events in a French study of 42 elderly institutionalized patients with T2D, with 243 events detected by CGM compared with only five events detected by SMBG (54). Furthermore, Bao et al. showed a greater improvement in time spent in range in elderly patients with T2D aged 65 years and older with CGM (47% at baseline vs 65% at 8 months) compared with SMBG (51% at baseline vs 49% at 8 months) (52).

Youths and young adults with T2D typically present with more aggressive disease, and use of CGM is needed for better outcomes in this subset of patients (55). A US pilot study in individuals aged 13 to 21 years with T2D using a variety of treatment regimens demonstrated that CGM increased the diabetes-related quality of life of patients, with a Pediatric Quality of Life Inventory score increase from 70 at baseline to 75 after 12 weeks (56). A further US pilot study in adolescent patients with T2D using basal-bolus insulin with metformin showed an HbA1c reduction of 2.8% in CGM users compared with SMBG (57). While all advisors agreed with continuous use of CGM in youth and young adults, it was also highlighted that it is necessary to stratify these patients; some may require intermittent use while others may need continuous CGM, depending on the severity of condition and the type and number of medications used.

Patients with T2D and cognitive impairment, dementia, or high stroke risk can also benefit from CGM. Patients with diabetes and dementia often have a higher risk of hypoglycemia. In a UK feasibility study of elderly patients with dementia or mental test score ≤8 and diabetes, of whom 92% had T2D and were using a variety of diabetes medications, caregivers expressed effectiveness of CGM and ease in monitoring of glucose levels (58). Hyperglycemia following a stroke is common in patients with and without diabetes (59), with CGM showing glucose levels to be significantly higher in those with diabetes (60). CGM can therefore be used to understand glycemic status as well as to determine the most beneficial treatment pathway in all patients with diabetes following a stroke (60).

Patients with T2D are at a greater risk of mental health impairment. CGM was associated with improved psychosocial health in patients with T2D using insulin (61, 62); therefore, it seems reasonable to deduce that patients with mental health impairment, such as depression or anxiety, would benefit from CGM use.

In renal transplant patients with a diabetes diagnosis, CGM was found to significantly reduce events of hyperglycemia and glucose levels in the 5 days following transplant compared with SMBG (63). Additionally, a retrospective chart review demonstrated reduced HbA1c of -0.73% in kidney transplant patients with T2D who were using CGM (64).

In patients with high cardiovascular risk, CGM is thought to improve management of diabetes. In a clinical trial of patients with T2D using non-insulin therapies and a high 10-year predicted atherosclerotic cardiovascular disease risk (24.0%), CGM use was associated with a significant decrease in atherosclerotic cardiovascular disease risk to 16.3% at 16 weeks, as well as a significant decrease in HbA1c, diabetes distress scale, cholesterol, and triglycerides (65). Furthermore, a Delphi consensus supported the use of CGM in all patients with diabetes and heart disease, especially with regard to the decrease in hypoglycemic and hyperglycemic events, enabling improved outcomes in these patients and the importance of CGM metrics for optimizing treatment (66).

Patients with cystic fibrosis-related diabetes have been shown to benefit from CGM, with a meta-analysis study showing a 0.4% lower HbA1c in CGM users compared with SMBG (67). Nevertheless, there is a need for studies directly comparing CGM and SMBG in those with cystic fibrosis-related diabetes.

Visual impairment in patients with T2D is thought to be approximately 10%. A survey of US healthcare professionals treating patients with diabetes and visual impairment, of which 79% of their patients were with T2D, reported a reduction in HbA1c greater than 0.5% in 87% of healthcare professionals and a reduction in hypoglycemia in 45% of healthcare professionals when using CGM with Siri for 6 months (68). Audible glucose alerts and voice activation were important features of CGM for patients with diabetes and visual impairment (68).

### 3.4 T2D patient profiles for intermittent use of CGM

Experts agreed on all statements relating to T2D patient profiles for intermittent use of CGM, with a level of agreement between 83.3% and 100% for the 8 statements (**Table 4**).

**Table 4:** Consensus statements on T2D patient profiles for intermittent use of CGM.

#	Statement	Consensus (%)
20	Intermittent use of CGM is strongly recommended for newly diagnosed T2D patients to support diabetes education and inform patients/healthcare practitioners about glycemic status.	83.34
21	Intermittent use of CGM is recommended for T2D patients undergoing intensification or deintensification of non-insulin therapies if continuous use is not possible for any reason.	91.67
22	Intermittent use of CGM is recommended for T2D patients who remain uncontrolled with their current non-insulin treatment regimens if continuous use is not possible for any reason.	100
23	Intermittent use of CGM is recommended for pregnant women with gestational diabetes if continuous use is not possible for any reason.	100
24	Intermittent use of CGM is recommended for transplant patients with T2D if continuous use is not possible for any reason.	100
25	Intermittent use of CGM may be recommended for T2D patients in the 14 days prior to visiting their doctor to provide insights into their glycemic status.	91.67
26	Intermittent use of CGM may be recommended for T2D patients taking therapies that may affect blood sugar levels (e.g. steroids) if continuous use is not possible for any reason.	91.67
27	Intermittent use of CGM may be recommended for T2D patients needing behavioral and lifestyle coaching if continuous use is not possible for any reason.	83.34

CGM, continuous glucose monitoring; T2D, type 2 diabetes.

Intermittent use of CGM has been shown to improve glycemic control compared with SMBG in a prospective trial of patients with T2D, with a 1% reduction in HbA1c versus 0.5% for SMBG (69). The intermittent use of CGM in newly diagnosed T2D is important to gauge baseline glycemic parameters, which will help to determine optimal therapy route as well as to assess the response to therapy (70, 71). Additionally, CGM in combination with glycemic minimization excursion was shown to significantly reduce HbA1c, with 67% of patients having a HbA1c less than 6.5% after 3 months, in newly diagnosed patients with T2D (72).

Patients undergoing intensification or deintensification of treatment require additional monitoring to avoid complications. CGM use in assisting such treatment changes in all patients with diabetes could lead to reduced risk of hospital admission and complications associated with high or low blood glucose, thereby reducing healthcare costs (70). A Canadian claims database study has shown that CGM assists in treatment changes and escalation in patients with T2D to a greater extent than SMBG, with a significant relative risk of treatment progression of 1.86 in non-insulin treatment-naïve patients and 2.03 in non-insulin treatment non-naïve patients (73). Additionally, in an Australian randomized controlled trial, rapid treatment intensification using CGM in T2D led to improved glycemia (74). Finally, a case series demonstrated that CGM data can facilitate clinical decisions regarding dose reductions of basal insulin (75).

Intermittent use of CGM can also be beneficial for patients with T2D who remain uncontrolled with their current treatment regimen. In a randomized controlled trial, intermittent use of CGM in patients with poorly controlled T2D using oral antidiabetics and/or insulin showed a significant decrease in HbA1c levels (-1.1%) compared with SMBG (-0.4%) at 12 weeks, as well as an improvement in diet and time spent exercising at 3 months (76). It is proposed that intermittent use of CGM in patients with T2D using oral antidiabetics can be used to determine whether the time in range is within the recommended guideline range (71).

Pregnant women with GDM could also benefit from intermittent use of CGM. In patients with GDM from Medina, Saudi Arabia, who used CGM for 3–7 days after GDM diagnosis as an educational tool, HbA1c was similar (5.7%) to those who only used SMBG (6.1%). However, mean glucose levels (6.1 to 5.1 mmol/l) and the standard deviation of mean glycemia (1.36 to 1.11 mmol/l) were reduced significantly in the CGM group (77). Additionally, in an observational study conducted in three hospitals in Saudi Arabia, a reduction in HbA1c in pregnant women with T2D of 0.71%, 1.2%, and 1.57% was observed at 3, 6, and 12 months, respectively, after starting CGM and a reduction of 0.81% and 1.3% was observed at 3 and 6 months, respectively, after starting CGM in patients with GDM who were using insulin (42).

Transplant patients with T2D are also candidates for intermittent CGM. Results from a prospective trial have supported the accuracy and feasibility of CGM in monitoring blood glucose levels in patients undergoing solid organ transplantation, of whom 35.4% had pre-existing T2D (78).

Furthermore, the use of CGM in patients in the 14 days prior to a doctors’ visit would enable the treating physician to understand patients’ glycemic patterns and HbA1c levels prior to next visit, facilitating dose or medication adjustments (71).

Certain therapies, especially steroids, are known to affect blood sugar levels. CGM can enable real-time monitoring of periods of medication-induced hyperglycemia (79). In non-insulin treated patients with T2D, CGM has been shown to facilitate in-depth evaluation of the effect of local steroids on glucose levels and events of severe hyperglycemia following steroid administration (80). Furthermore, intermittent CGM can facilitate modifications to insulin dosing during episodes of severe hyperglycemia caused by steroids (71).

CGM can also be used for behavioral and lifestyle coaching in patients with T2D, since patients are able to see the real-time effects of lifestyle choices on their blood glucose levels. In a retrospective study of professional CGM use in patients with T2D using a variety of treatment regimens, of which over half were using non-intensive regimens, nearly all patients made changes to their diet and exercise with CGM (81). Additionally, in a US pilot study of patients with T2D using non-insulin therapies, significant increases in healthy eating were reported with the use of CGM alongside diabetes self-management education (82). Finally, a randomized controlled trial of patients with T2D using non-insulin therapies demonstrated that physical activity counseling, including feedback from CGM devices, led to a decrease in BMI and light or sedentary activity, an increase in moderate activity, and lower relapse rates after 8 weeks (83).

## 4 Discussion

This consensus provides 27 statements relating to the use of CGM in patients with T2D in Saudi Arabia. The expert panel generated statements on the background of T2D in KSA and definitions of intensive and non-intensive management and have made recommendations for general use of CGM in T2D as well as specific patient profiles for continuous use and intermittent use of CGM. Local and international evidence was summarized for each of the statements and local expert opinion provided based on current clinical practices and experience.

These statements align with the diabetes standards of care or guidelines from the US (10, 11), UK (12) and Asia (13) whilst focusing on T2D patients who are non-intensively managed. The experts strongly recommend CGM for T2D patients at risk of hypoglycemia, which is similar to AACE and NICE standards of care, where CGM is recommended in patients with problematic hypoglycemia and severe or recurrent hypoglycemia, respectively (11, 12). Additionally, the experts strongly recommend CGM for patients using basal or premixed insulin; this is comparable to the AACE standards of care, which states that CGM may also be recommended in patients with T2D on less intensive insulin therapy (11). Whilst the experts from this consensus recommend CGM for patients with cognitive or mental health impairment, NICE recommends for those who require help with glucose monitoring due to disability or other condition (12). Furthermore, the experts recommend CGM for elderly or frail T2D patients with multiple comorbidities, similar to the APAC consensus, which recommends it for frail patients aged 65 years or older (13). Additionally, experts recommend intermittent use of CGM for pregnant women with gestational diabetes, if continuous use is not possible for any reason. Similarly, AACE specifies that CGM may also be recommended in women with GDM who are not using insulin (11). Additional non-intensively managed T2D patient profiles recommended for continuous CGM in this consensus include youths or young adults, those with dementia or high-risk stroke, those with renal transplant, high cardiovascular risk, CF-related diabetes and those suffering from blindness.

Whilst many of these diabetes standards of care or guidelines state that continuous or intermittent use of CGM can be undertaken, this consensus provides recommendations for specific patient profiles for intermittent use of CGM, a strategy which can provide the benefits of CGM use where resources might be limited (84). Intermittent CGM has been shown to be beneficial in certain situations, facilitating patients in gaining a deeper understanding of the impact of diet and activity as well as making healthy lifestyle choices (85).

Statements within this consensus are likely generalizable, particularly to countries within the Middle East region with similar healthcare systems and patient populations. Whilst much of the evidence supporting these consensus statements is from international studies rather than local studies, the experience of the expert panel reflected the findings from these studies. Further research is needed on the use of CGM in the non-intensively managed T2D population in KSA.

## 5 Conclusions

In this consensus, evidence from local and international studies as well as expert opinion has been used to build a framework for the use of CGM in patients with T2D who are non-intensively managed in KSA. In order to expand the use of CGM into the wider population of T2D and enable such individuals to benefit from this technology, there is the need for a shift in healthcare services, education, and attitudes across the country.

## Data Availability

The raw data supporting the conclusions of this article will be made available by the authors, without undue reservation.

## Glossary

AACE: American Association of Clinical Endocrinologists
ADA: American Diabetes Association
APAC: Asia-Pacific
CGM: Continuous Glucose Monitoring
FGM: flash glucose monitoring
GDM: Gestational diabetes mellitus
HbA1c: Hemoglobin A1c
isCGM: intermittently scanned continuous glucose monitoring
KSA: Kingdom of Saudi Arabia
MDI: multiple daily injections
NICE: UK National Institute for Health and Care Excellence
SMBG: Self-monitoring of blood glucose
T1D: Type 1 diabetes
T2D: Type 2 diabetes

## References

[1] International Diabetes Federation . Idf Diabetes Atlas, 10th Edition. (2021) Brussels, Belgium: International Diabetes Federation.

[2] Jarrar M Abusalah MAH Albaker W Al-Bsheish M Alsyouf A Al-Mugheed K . Prevalence of type 2 diabetes mellitus in the general population of Saudi Arabia, 2000-2020: A systematic review and meta-analysis of observational studies. Saudi J Med Med Sci. (2023) 11:1–10. doi: 10.4103/sjmms.sjmms_394_22, PMID: 36909010 PMC9997860

[3] Holzer R Bloch W Brinkmann C . Continuous glucose monitoring in healthy adults-possible applications in health care, wellness, and sports. Sensors. (2022) 22:2030. doi: 10.3390/s22052030, PMID: 35271177 PMC8915088

[4] Galindo RJ Aleppo G . Continuous glucose monitoring: the achievement of 100 years of innovation in diabetes technology. Diabetes Res Clin Pract. (2020) 170:108502. doi: 10.1016/j.diabres.2020.108502, PMID: 33065179 PMC7736459

[5] Klonoff DC Ahn D Drincic A . Continuous glucose monitoring: A review of the technology and clinical use. Diabetes Res Clin Pract. (2017) 133:178–92. doi: 10.1016/j.diabres.2017.08.005, PMID: 28965029

[6] Polonsky WH Fortmann AL . Impact of real-time continuous glucose monitoring data sharing on quality of life and health outcomes in adults with type 1 diabetes. Diabetes Technol Ther. (2021) 23:195–202. doi: 10.1089/dia.2020.0466, PMID: 32991199 PMC7906862

[7] Lin R Brown F James S Jones J Ekinci E . Continuous glucose monitoring: A review of the evidence in type 1 and 2 diabetes mellitus. Diabetes Med. (2021) 38:e14528. doi: 10.1111/dme.14528, PMID: 33496979

[8] Mayberry LS Guy C Hendrickson CD McCoy AB Elasy T . Rates and correlates of uptake of continuous glucose monitors among adults with type 2 diabetes in primary care and endocrinology settings. J Gen Intern Med. (2023) 38:2546–52. doi: 10.1007/s11606-023-08222-3, PMID: 37254011 PMC10228889

[9] Lacy ME Lee KE Atac O Heier K Fowlkes J Kucharska-Newton A . Patterns and trends in continuous glucose monitoring utilization among commercially insured individuals with type 1 diabetes: 2010–2013 to 2016–2019. Clin Diabetes. (2024) 42:388–97. doi: 10.2337/cd23-0051, PMID: 39015169 PMC11247039

[10] ElSayed NA Aleppo G Aroda VR Bannuru RR Brown FM Bruemmer D . 7. Diabetes technology: standards of care in diabetes-2023. Diabetes Care. (2023) 46:S111–S27. doi: 10.2337/dc23-S007, PMID: 36507635 PMC9810474

[11] Grunberger G Sherr J Allende M Blevins T Bode B Handelsman Y . American association of clinical endocrinology clinical practice guideline: the use of advanced technology in the management of persons with diabetes mellitus. Endocr Pract. (2021) 27:505–37. doi: 10.1016/j.eprac.2021.04.008, PMID: 34116789

[12] National Institute for Health and Care Excellence . Type 2 Diabetes in Adults: Management. (2022) London, UK: National Institute for Health and Care Excellence.

[13] Kong APS Lim S Yoo SH Ji L Chen L Bao Y . Asia-Pacific consensus recommendations for application of continuous glucose monitoring in diabetes management. Diabetes Res Clin Pract. (2023) 201:110718. doi: 10.1016/j.diabres.2023.110718, PMID: 37196707

[14] Al Mansour MA . The prevalence and risk factors of type 2 diabetes mellitus (Dmt2) in a semi-urban Saudi population. Int J Environ Res Public Health. (2019) 17:7. doi: 10.3390/ijerph17010007, PMID: 31861311 PMC6981763

[15] Al-Daghri NM Al-Attas OS Alokail MS Alkharfy KM Yousef M Sabico SL . Diabetes mellitus type 2 and other chronic non-communicable diseases in the central region, Saudi Arabia (Riyadh cohort 2): A decade of an epidemic. BMC Med. (2011) 9:76. doi: 10.1186/1741-7015-9-76, PMID: 21689399 PMC3141541

[16] Al-Esawi H Amer SA . Prevalence of complications among Saudi males type 2 diabetic patients in Riyadh primary health care centers, 2019. Diabetes Updates. (2021) 7:1–11. doi: 10.15761/DU.1000158

[17] Alamer WM Qutub RM Alsaloumi EA Natto NK Alshehri RM Khafagy A . Prevalence of sleep disorders among patients with type 2 diabetes mellitus in Makkah City: A cross-sectional study. Cureus. (2022) 14:e33088. doi: 10.7759/cureus.33088, PMID: 36721622 PMC9884065

[18] Alshaya OA Korayem GB Alghwainm M Alyami W Alotaibi A Alyami MS . The prevalence of cardiovascular diseases, chronic kidney disease, and obesity in patients with type 2 diabetes mellitus and the description of concurrent treatments: A two-center retrospective cross-sectional study in Saudi Arabia. Saudi Pharm J. (2024) 32:102054. doi: 10.1016/j.jsps.2024.102054, PMID: 38590611 PMC10999870

[19] Alsenany S Al Saif A . Incidence of Diabetes Mellitus Type 2 Complications among Saudi Adult Patients at Primary Health Care Center. J Phys Ther Sci. (2015) 27:1727–30. doi: 10.1589/jpts.27.1727, PMID: 26180307 PMC4499970

[20] Salman RA Al-Rubeaan KA . Incidence and risk factors of hypertension among Saudi type 2 diabetes adult patients: an 11-year prospective randomized study. J Diabetes Complications. (2009) 23:95–101. doi: 10.1016/j.jdiacomp.2007.10.004, PMID: 18413199

[21] Alramadan MJ Magliano DJ Alhamrani HA Alramadan AJ Alameer SM Amin GM . Lifestyle factors and macro- and micro-vascular complications among people with type 2 diabetes in Saudi Arabia. Diabetes Metab Syndr. (2019) 13:484–91. doi: 10.1016/j.dsx.2018.11.007, PMID: 30641750

[22] Alzaheb RA Altemani AH . Prevalence and associated factors of dyslipidemia among adults with type 2 diabetes mellitus in Saudi Arabia. Diabetes Metab Syndr Obes. (2020) 13:4033–40. doi: 10.2147/DMSO.S246068, PMID: 33149642 PMC7604430

[23] AlShahrani MS . Prevalence of obesity and overweight among type 2 diabetic patients in Bisha, Saudi Arabia. J Family Med Prim Care. (2021) 10:143–8. doi: 10.4103/jfmpc.jfmpc_1349_20, PMID: 34017717 PMC8132811

[24] Mugharbel KM Al-Mansouri MA . Prevalence of obesity among type 2 diabetic patients in Al-Khobar primary health care centers. J Family Community Med. (2003) 10:49–53. doi: 10.4103/2230-8229.97856, PMID: 23011992 PMC3425767

[25] Al-Rubeaan K Youssef AM Subhani SN Ahmad NA Al-Sharqawi AH Al-Mutlaq HM . Diabetic nephropathy and its risk factors in a society with a type 2 diabetes epidemic: A Saudi national diabetes registry-based study. PLoS One. (2014) 9:e88956. doi: 10.1371/journal.pone.0088956, PMID: 24586457 PMC3931705

[26] Aljehani EA Alhawiti AE Mohamad RM . Prevalence and determinants of diabetic retinopathy among type 2 diabetic patients in Saudi Arabia: A systematic review. Cureus. (2023) 15:e42771. doi: 10.7759/cureus.42771, PMID: 37663987 PMC10469098

[27] Al Ayed MY Ababneh M Robert AA Al-Musalum M Sabrery D Amer M . Prevalence and risk factors for diabetic peripheral neuropathy among patients with type 2 diabetes in Saudi Arabia: A cross-sectional study. Curr Diabetes Rev. (2023) 19:e141122210875. doi: 10.2174/1573399819666221114105817, PMID: 36380415

[28] Jatooi NA Alsulaiman ASA Alromaih NJ Abdullah Albahrani B Alkhattaf IM Alyami F . Prevalence of diabetic peripheral neuropathy among type ii diabetic patients in King Fahd university hospital, Khobar, Kingdom of Saudi Arabia. Hosp Pract. (2021) 49:63–70. doi: 10.1080/21548331.2020.1853995, PMID: 33216654

[29] Ponirakis G Elhadd T Al Ozairi E Brema I Chinnaiyan S Taghadom E . Prevalence and risk factors for diabetic peripheral neuropathy, neuropathic pain and foot ulceration in the Arabian gulf region. J Diabetes Investig. (2022) 13:1551–9. doi: 10.1111/jdi.13815, PMID: 35445568 PMC9434582

[30] Alzahrani A Alghamdi A Alqarni T Alshareef R Alzahrani A . Prevalence and predictors of depression, anxiety, and stress symptoms among patients with type ii diabetes attending primary healthcare centers in the Western region of Saudi Arabia: A cross-sectional study. Int J Ment Health Syst. (2019) 13:48. doi: 10.1186/s13033-019-0307-6, PMID: 31341512 PMC6631923

[31] Alzughbi T Badedi M Darraj H Hummadi A Jaddoh S Solan Y . Diabetes-related distress and depression in Saudis with type 2 diabetes. Psychol Res Behav Manag. (2020) 13:453–8. doi: 10.2147/PRBM.S255631, PMID: 32547267 PMC7239888

[32] Algeffari M Alkhamis A Almesned A Alghammas N Albulayhi S AlGoblan A . Obstructive sleep apnea among people with type 2 diabetes in Saudi Arabia: A cross-sectional study. Majmaah J Heal Sci. (2018) 6:32–9. doi: 10.5455/mjhs.2018.02.005

[33] Kalan Farmanfarma KH Ansari-Moghaddam A Zareban I Adineh HA . Prevalence of type 2 diabetes in middle-east: systematic review& Meta-analysis. Prim Care Diabetes. (2020) 14:297–304. doi: 10.1016/j.pcd.2020.01.003, PMID: 32044288

[34] Delahanty LM Halford BN . The role of diet behaviors in achieving improved glycemic control in intensively treated patients in the diabetes control and complications trial. Diabetes Care. (1993) 16:1453–8. doi: 10.2337/diacare.16.11.1453, PMID: 8299434

[35] Al Hayek AA Al Dawish MA . Use of flash glucose monitoring and glycemic control in patients with type 2 diabetes mellitus not treated with an intensive insulin regimen: 1-year real-life retrospective cohort study. Adv Ther. (2023) 40:2855–68. doi: 10.1007/s12325-023-02508-y, PMID: 37133646

[36] Ajjan RA Battelino T Cos X Del Prato S Philips JC Meyer L . Continuous glucose monitoring for the routine care of type 2 diabetes mellitus. Nat Rev Endocrinol. (2024) 20:426–40. doi: 10.1038/s41574-024-00973-1, PMID: 38589493

[37] Aleppo G Hirsch IB Parkin CG McGill J Galindo R Kruger DF . Coverage for continuous glucose monitoring for individuals with type 2 diabetes treated with nonintensive therapies: an evidence-based approach to policymaking. Diabetes Technol Ther. (2023) 25:741–51. doi: 10.1089/dia.2023.0268, PMID: 37471068 PMC10611973

[38] Cowart K Updike WH Franks R . Continuous glucose monitoring in persons with type 2 diabetes not using insulin. Expert Rev Med Devices. (2021) 18:1049–55. doi: 10.1080/17434440.2021.1992274, PMID: 34633261

[39] Wright EE Subramanian S . Evolving use of continuous glucose monitoring beyond intensive insulin treatment. Diabetes Technol Ther. (2021) 23:S12–S8. doi: 10.1089/dia.2021.0191, PMID: 34546082

[40] Kwon SY Moon JS . Advances in continuous glucose monitoring: clinical applications. Endocrinol Metab (Seoul). (2025) 40:161–73. doi: 10.3803/EnM.2025.2370, PMID: 40195726 PMC12061739

[41] Behnke A Lucas KS Parkin CG Reddy V Orlowski M Edwards A . Persistent performance improvement using team resources and continuous glucose monitoring in patients with poorly controlled type 2 diabetes. Diabetes Spectr. (2025) 38:353–8. doi: 10.2337/ds24-0085, PMID: 40823609 PMC12357217

[42] Alsifri S Almalki A Alsifri S Alsifri N Moatism M Alsifri S . The impact of the freestyle libretm flash glucose monitoring system on glycemic control in patients with diabetes; observational multicenter 15-months study. Int J Clin Med. (2022) 13:391–404. doi: 10.4236/ijcm.2022.138027

[43] Al Hayek A Robert AA Al Dawish M . Impact of the freestyle libre flash glucose monitoring system on diabetes- self-management practices and glycemic control among patients with type 2 diabetes in Saudi Arabia: A prospective study. Diabetes Metab Syndr. (2021) 15:557–63. doi: 10.1016/j.dsx.2021.02.027, PMID: 33689937

[44] Ferreira ROM Trevisan T Pasqualotto E Chavez MP Marques BF Lamounier RN . Continuous glucose monitoring systems in noninsulin-treated people with type 2 diabetes: A systematic review and meta-analysis of randomized controlled trials. Diabetes Technol Ther. (2024) 26:252–62. doi: 10.1089/dia.2023.0390, PMID: 38090767

[45] Gurajala S . Healthcare system in the kingdom of Saudi Arabia: an expat doctor’s perspective. Cureus. (2023) 15:e38806. doi: 10.7759/cureus.38806, PMID: 37303448 PMC10250784

[46] Oser TK Hall TL Dickinson LM Callen E Carroll JK Nease DE Jr. . Continuous glucose monitoring in primary care: understanding and supporting clinicians’ Use to enhance diabetes care. Ann Fam Med. (2022) 20:541–7. doi: 10.1370/afm.2876, PMID: 36443083 PMC9705045

[47] Martens TW . Continuous glucose monitoring in primary care - are we there? Curr Opin Endocrinol Diabetes Obes. (2022) 29:10–6. doi: 10.1097/MED.0000000000000689, PMID: 34845158

[48] Silbert R Salcido-Montenegro A Rodriguez-Gutierrez R Katabi A McCoy RG . Hypoglycemia among patients with type 2 diabetes: epidemiology, risk factors, and prevention strategies. Curr Diabetes Rep. (2018) 18:53. doi: 10.1007/s11892-018-1018-0, PMID: 29931579 PMC6117835

[49] Elshebiny AM Alali HA Alamer ZM Alsultan YK Alkhalaf HE Alkishi AM . The incidence of hypoglycemia and its risk factors among diabetic patients in the eastern province of Saudi Arabia. IJMDC. (2021) 5:614–21. doi: 10.24911/IJMDC.51-1609148506

[50] Surrati AMQ Alanazi AA Bukhari SS Alfadhli EM . Hypoglycemia unawareness among insulin-treated diabetic patients in madinah, Saudi Arabia: prevalence and risk factors. Front Endocrinol. (2023) 14:1239524. doi: 10.3389/fendo.2023.1239524, PMID: 37964960 PMC10640969

[51] AlKhaldi YM AlKhaldi AY AlQahtani AS Al-Shahrani BS Meshawi EA Albishri BM . Incidence of hypoglycemia and its risk factors among diabetics during Ramadan in Abha City, Aseer Region, Ksa. J Family Med Prim Care. (2019) 8:2793–8. doi: 10.4103/jfmpc.jfmpc_250_19, PMID: 31681644 PMC6820420

[52] Bao S Bailey R Calhoun P Beck RW . Effectiveness of continuous glucose monitoring in older adults with type 2 diabetes treated with basal insulin. Diabetes Technol Ther. (2022) 24:299–306. doi: 10.1089/dia.2021.0494, PMID: 34939824 PMC9127838

[53] Martens T Beck RW Bailey R Ruedy KJ Calhoun P Peters AL . Effect of continuous glucose monitoring on glycemic control in patients with type 2 diabetes treated with basal insulin: A randomized clinical trial. JAMA. (2021) 325:2262–72. doi: 10.1001/jama.2021.7444, PMID: 34077499 PMC8173473

[54] Bouillet B Tscherter P Vaillard L Nonciaux C Hourdain P Ravier A . Frequent and severe hypoglycaemia detected with continuous glucose monitoring in older institutionalised patients with diabetes. Age Ageing. (2021) 50:2088–93. doi: 10.1093/ageing/afab128, PMID: 34324624

[55] Chan CL . Use of continuous glucose monitoring in youth-onset type 2 diabetes. Curr Diabetes Rep. (2017) 17:66. doi: 10.1007/s11892-017-0905-0, PMID: 28726154

[56] Chesser H Srinivasan S Puckett C Gitelman SE Wong JC . Real-time continuous glucose monitoring in adolescents and young adults with type 2 diabetes can improve quality of life. J Diabetes Sci Technol. (2024) 18:911–9. doi: 10.1177/19322968221139873, PMID: 36416098 PMC11307231

[57] Chang N Barber ROB Llovido Alula J Durazo-Arvizu R Chao LC . Continuous glucose monitoring versus standard of care in adolescents with type 2 diabetes: A pilot randomized cross-over trial. J Diabetes Sci Technol. (2023) 17:1419–20. doi: 10.1177/19322968231178284, PMID: 37278187 PMC10563525

[58] Mattishent K Lane K Salter C Dhatariya K May HM Neupane S . Continuous glucose monitoring in older people with diabetes and memory problems: A mixed-methods feasibility study in the UK. BMJ Open. (2019) 9:e032037. doi: 10.1136/bmjopen-2019-032037, PMID: 31740472 PMC6937046

[59] Radermecker RP Scheen AJ . Management of blood glucose in patients with stroke. Diabetes Metab. (2010) 36 Suppl 3:S94–9. doi: 10.1016/S1262-3636(10)70474-2, PMID: 21211743

[60] Allport L Baird T Butcher K Macgregor L Prosser J Colman P . Frequency and temporal profile of poststroke hyperglycemia using continuous glucose monitoring. Diabetes Care. (2006) 29:1839–44. doi: 10.2337/dc06-0204, PMID: 16873789

[61] Crawford MA Hicks C Zhou T Kleinhanzl A Singh H . 63-lb: real-time continuous glucose monitoring (Rtcgm) is associated with improved clinical and psychosocial health in people with type 2 diabetes on basal insulin (T2d-Bi). Diabetes Care. (2023) 72:63–LB. doi: 10.2337/db23-63-LB

[62] Soriano EC Polonsky WH . The influence of real-time continuous glucose monitoring on psychosocial outcomes in insulin-using type 2 diabetes. J Diabetes Sci Technol. (2023) 17:1614–22. doi: 10.1177/19322968221094831, PMID: 35533137 PMC10658676

[63] Jandovitz N George SJ Abate M Kressel AM Bolognese AC Lau L . A randomized trial of continuous glucose monitoring to improve post-transplant glycemic control. Clin Transplant. (2023) 37:e15139. doi: 10.1111/ctr.15139, PMID: 37725341

[64] Agate W Pham N-Y Roumelioti M-E Argyropoulos C . Glycemic control with continuous glucose monitoring in kidney transplant patients: Pub326. J Am Soc Nephrol. (2022) 33:975. doi: 10.1681/ASN.20223311S1975c

[65] Reed J Dong T Eaton E Friswold J Porges J Al-Kindi SG . Continuous glucose monitoring for glycaemic control and cardiovascular risk reduction in patients with type 2 diabetes not on insulin therapy: A clinical trial. Diabetes Obes Metab. (2024) 26:2881–9. doi: 10.1111/dom.15608, PMID: 38680050

[66] Di Mario C Genovese S Lanza GA Mannucci E Marenzi G Sciatti E . Role of continuous glucose monitoring in diabetic patients at high cardiovascular risk: an expert-based multidisciplinary Delphi consensus. Cardiovasc Diabetol. (2022) 21:164. doi: 10.1186/s12933-022-01598-2, PMID: 36030229 PMC9420264

[67] Kumar S Soldatos G Ranasinha S Teede H Pallin M . Continuous glucose monitoring versus self-monitoring of blood glucose in the management of cystic fibrosis related diabetes: A systematic review and meta-analysis. J Cyst Fibros. (2023) 22:39–49. doi: 10.1016/j.jcf.2022.07.013, PMID: 35906171

[68] Akturk HK Snell-Bergeon J Shah VN . Health care professionals’ Perspectives on use of diabetes technologies for managing visually impaired patients with diabetes. J Diabetes Sci Technol. (2023) 17:1610–3. doi: 10.1177/19322968221101629, PMID: 35658590 PMC10658667

[69] Ehrhardt NM Chellappa M Walker MS Fonda SJ Vigersky RA . The effect of real-time continuous glucose monitoring on glycemic control in patients with type 2 diabetes mellitus. J Diabetes Sci Technol. (2011) 5:668–75. doi: 10.1177/193229681100500320, PMID: 21722581 PMC3192632

[70] Klupa T Czupryniak L Dzida G Fichna P Jarosz-Chobot P Gumprecht J . Expanding the role of continuous glucose monitoring in modern diabetes care beyond type 1 disease. Diabetes Ther. (2023) 14:1241–66. doi: 10.1007/s13300-023-01431-3, PMID: 37322319 PMC10299981

[71] Saboo B Unnikrishnan R Kesavadev J Tiwaskar M Czupryniak L Chawla M . Intermittent use of continuous glucose monitoring: A new paradigm in treatment of type 2 diabetes. J Assoc Physicians India. (2023) 71:11–2. doi: 10.5005/japi-11001-0274, PMID: 37355844

[72] Oser TK Cucuzzella M Stasinopoulos M Moncrief M McCall A Cox DJ . An innovative, paradigm-shifting lifestyle intervention to reduce glucose excursions with the use of continuous glucose monitoring to educate, motivate, and activate adults with newly diagnosed type 2 diabetes: pilot feasibility study. JMIR Diabetes. (2022) 7:e34465. doi: 10.2196/34465, PMID: 35050857 PMC8908197

[73] Harris SB Levrat-Guillen F . Use of the freestyle libre system and diabetes treatment progression in T2dm: results from a retrospective cohort study using a Canadian private payer claims database. Diabetes Obes Metab. (2023) 25:1704–13. doi: 10.1111/dom.15025, PMID: 36811267

[74] Middleton TL Constantino MI McGill M D’Souza M Yue DK Twigg SM . Improving beta-cell secretory function and glycaemia in young-onset type 2 diabetes: A pilot, 12-month, randomized trial of a novel, continuous glucose monitor-guided, rapid treatment intensification strategy incorporating empagliflozin and liraglutide. Diabetes Obes Metab. (2022) 24:747–51. doi: 10.1111/dom.14621, PMID: 34882926

[75] Ehrhardt NM Aroda VR Galindo RJ Peters AL Shubrook JH . Use of continuous glucose monitoring and glucagon-like peptide 1 receptor agonist therapy to achieve individualized treatment goals in insulin-treated people with type 2 diabetes: A case series and expert opinion. Clin Diabetes. (2024) 42:341–50. doi: 10.2337/cd23-0047, PMID: 38666194 PMC11040021

[76] Yoo HJ An HG Park SY Ryu OH Kim HY Seo JA . Use of a real time continuous glucose monitoring system as a motivational device for poorly controlled type 2 diabetes. Diabetes Res Clin Pract. (2008) 82:73–9. doi: 10.1016/j.diabres.2008.06.015, PMID: 18701183

[77] Alfadhli E Osman E Basri T . Use of a real time continuous glucose monitoring system as an educational tool for patients with gestational diabetes. Diabetol Metab Syndr. (2016) 26:48. doi: 10.1186/s13098-016-0161-5, PMID: 27468313 PMC4962392

[78] Voglova Hagerf B Protus M Nemetova L Mraz M Kieslichova E Uchytilova E . Accuracy and feasibility of real-time continuous glucose monitoring in critically ill patients after abdominal surgery and solid organ transplantation. Diabetes Care. (2024) 47:956–63. doi: 10.2337/dc23-1663, PMID: 38412005 PMC11116916

[79] Jain AB Lai V . Medication-induced hyperglycemia and diabetes mellitus: A review of current literature and practical management strategies. Diabetes Ther. (2024) 15:2001–25. doi: 10.1007/s13300-024-01628-0, PMID: 39085746 PMC11330434

[80] Safran O Fraind-Maya G Kandel L Leibowitz G Beyth S . The effect of steroid injection into the shoulder on glycemia in patients with type 2 diabetes. JSES Int. (2022) 6:843–8. doi: 10.1016/j.jseint.2022.05.016, PMID: 36081707 PMC9446203

[81] Kesavadev J Vigersky R Shin J Pillai PBS Shankar A Sanal G . Assessing the therapeutic utility of professional continuous glucose monitoring in type 2 diabetes across various therapies: A retrospective evaluation. Adv Ther. (2017) 34:1918–27. doi: 10.1007/s12325-017-0576-x, PMID: 28667580

[82] Polonsky WH Fortmann AL Soriano EC Guzman SJ Funnell MM . The Ah-Ha! Project: transforming group diabetes self-management education through the addition of flash glucose monitoring. Diabetes Technol Ther. (2023) 25:194–200. doi: 10.1089/dia.2022.0419, PMID: 36409486

[83] Allen NA Fain JA Braun B Chipkin SR . Continuous glucose monitoring counseling improves physical activity behaviors of individuals with type 2 diabetes: A randomized clinical trial. Diabetes Res Clin Pract. (2008) 80:371–9. doi: 10.1016/j.diabres.2008.01.006, PMID: 18304674 PMC2430041

[84] Ziegler R Heinemann L Freckmann G Schnell O Hinzmann R Kulzer B . Intermittent use of continuous glucose monitoring: expanding the clinical value of Cgm. J Diabetes Sci Technol. (2021) 15:684–94. doi: 10.1177/1932296820905577, PMID: 32064909 PMC8120049

[85] Bendixen BE Wilhelmsen-Langeland A Lomborg K Måkestad R Iversen MM Søfteland E . Intermittent use of continuous glucose monitoring in type 2 diabetes is preferred: A qualitative study of patients’ Experiences. Sci Diabetes Self-Management Care. (2025) 51:323–32. doi: 10.1177/26350106251326517, PMID: 40116013 PMC12127601

